# A glucose-supplemented diet enhances gut barrier integrity in *Drosophila*

**DOI:** 10.1242/bio.056515

**Published:** 2021-03-08

**Authors:** Anthony Galenza, Edan Foley

**Affiliations:** 1 Department of Medical Microbiology and Immunology, University of Alberta, Edmonton, AB T6G 2S2, Canada; 2 Department of Molecular and Cellular Physiology, Stanford University School of Medicine, Stanford, CA, 94305, USA

**Keywords:** Aging, *Drosophila melanogaster*, Longevity, Glucose, Tight Junctions

## Abstract

Dietary intervention has received considerable attention as an approach to extend lifespan and improve aging. However, questions remain regarding optimal dietary regimes and underlying mechanisms of lifespan extension. Here, we asked how an increase of glucose in a chemically defined diet extends the lifespan of adult *Drosophila* *melanogaster*. We showed that glucose-dependent lifespan extension is not a result of diminished caloric intake, or changes to systemic insulin activity, two commonly studied mechanisms of lifespan extension. Instead, we found that flies raised on glucose-supplemented food increased the expression of cell-adhesion genes, delaying age-dependent loss of intestinal barrier integrity. Furthermore, we showed that chemical disruption of the gut barrier negated the lifespan extension associated with glucose treatment, suggesting that glucose-supplemented food prolongs adult viability by enhancing the intestinal barrier. We believe our data contribute to understanding intestinal homeostasis, and may assist efforts to develop preventative measures that limit effects of aging on health.

## Abstract (precis)

**Summary:** How diet affects lifespan has received much attention. We found that maintenance of intestinal barrier integrity is likely an important factor in glucose-dependent lifespan extension in flies.

## INTRODUCTION

As nutrition has established impacts on health, optimizing feeding regimes to promote healthy aging has received considerable attention (Kalache et al., 2019). Nutritional deficiencies increase risk of developing a number of age-related chronic diseases, but we have limited understanding of dietary interventions that counter age-dependent deterioration of tissue and organ function (Shlisky et al., 2017). Model organisms, including *Drosophila melanogaster*, are excellent tools to study interactions between nutrition and organ function with age (Fontana and Partridge, 2015; Lee et al., 2015; Piper and Partridge, 2017). Flies are a genetically tractable system that uses evolutionarily conserved pathways such as the insulin and TOR responses to control nutrient sensing, acquisition, and use. Importantly, researchers can grow flies on chemically defined holidic media that allow investigators to quantify effects of macronutrients on health and lifespan (Piper et al., 2014). Nutritional geometry work emphasized the importance of relative macronutrient levels for *Drosophila* fitness, and revealed that low protein-to-carbohydrate ratios extend longevity, with maximal benefits at approximately a 1:16 protein:carbohydrate ratio (Lee et al., 2008; Simpson and Raubenheimer, 2009; Solon-Biet et al., 2015). Notably, low protein-to-carbohydrate ratios also extend longevity in mice (Solon-Biet et al., 2014), suggesting a conserved effect of protein-to-carbohydrate ratios on animal lifespan.

Work with flies has provided mechanistic insights into the deleterious consequences of excess carbohydrates (Graham and Pick, 2017). For example, flies raised on a high-sucrose diet (1.0 M compared to 0.15 M controls) have increased weight, alongside elevated triglyceride stores, and insulin resistance (Musselman et al., 2011). High-sucrose treatment (1.0 M compared to 0.15 M controls) reduces *Drosophila* lifespan (Na et al., 2013), even with transient exposure (1.2 M compared to 0.15 M controls) in young adults (Dobson et al., 2017). However, supplementation with water rescues survival during high-sucrose treatment, while weight and insulin activity remain affected, suggesting that dehydration rather than increased sugar levels reduces lifespan (van Dam et al., 2020). Conversely, decreased sucrose supplementation (0–5 mM compared to 50 mM controls) reduces median lifespan in female flies raised on a holidic diet, while higher levels of sucrose (75–100 mM) have no effect (Wu et al., 2020). On a synthetic diet, higher sucrose (5.3% compared to 1.3%) extends median lifespan of *Oregon-R* females (Reis, 2016). Recently, we found that addition of 0.56 M glucose to a holidic medium that contains 0.05 M sucrose extends *Drosophila* lifespan through an unknown mechanism (Galenza et al., 2016). Here, we used a combination of genomic, cellular, and metabolic assays to suggest possible roles for the intestinal epithelial barrier in glucose-dependent extension of longevity.

As flies age, organization of the intestinal epithelium breaks down, and the intestine fails as a barrier to extrinsic factors (Biteau et al., 2008; Choi et al., 2008; Park et al., 2009). Intestinal epithelial deterioration is a consistent characteristic of aging in model organisms, including *C. elegans* (Gelino et al., 2016), zebrafish (Dambroise et al., 2016), mice (Thevaranjan et al., 2017), and even primates (Mitchell et al., 2017). Evidence suggests that the human intestinal barrier also appears to weaken with age (Mabbott, 2015). As nutrition affects both lifespan and barrier integrity, it is possible that diets that extend lifespan, such as those with a low protein-to-carbohydrate ratio, do so, at least in part, through improved intestinal barrier maintenance. However, how sugar affects both the intestinal barrier and lifespan remains poorly understood.

In this study, we found that glucose supplementation extends lifespan without diminishing caloric intake, or lowering systemic insulin activity. Instead, we showed that glucose-supplemented food extends the lifespan of adult *Drosophila* in conjunction with improved intestinal barrier integrity in aging flies. Glucose-treated flies have increased expression of cell junction genes and higher levels of the septate junction protein Coracle localized at intestinal bicellular junctions, and flies raised on glucose-supplemented food maintain barrier function to a later age than their control counterparts. Notably, chemical disruption of the epithelial barrier counters the benefits associated with culture on glucose-supplemented food. Combined, our data implicate regulation of gut barrier integrity in glucose-dependent extension of *Drosophila* longevity.

## RESULTS

### Glucose-supplemented holidic food promotes maintenance of energy stores with age

**Fig. 1.**
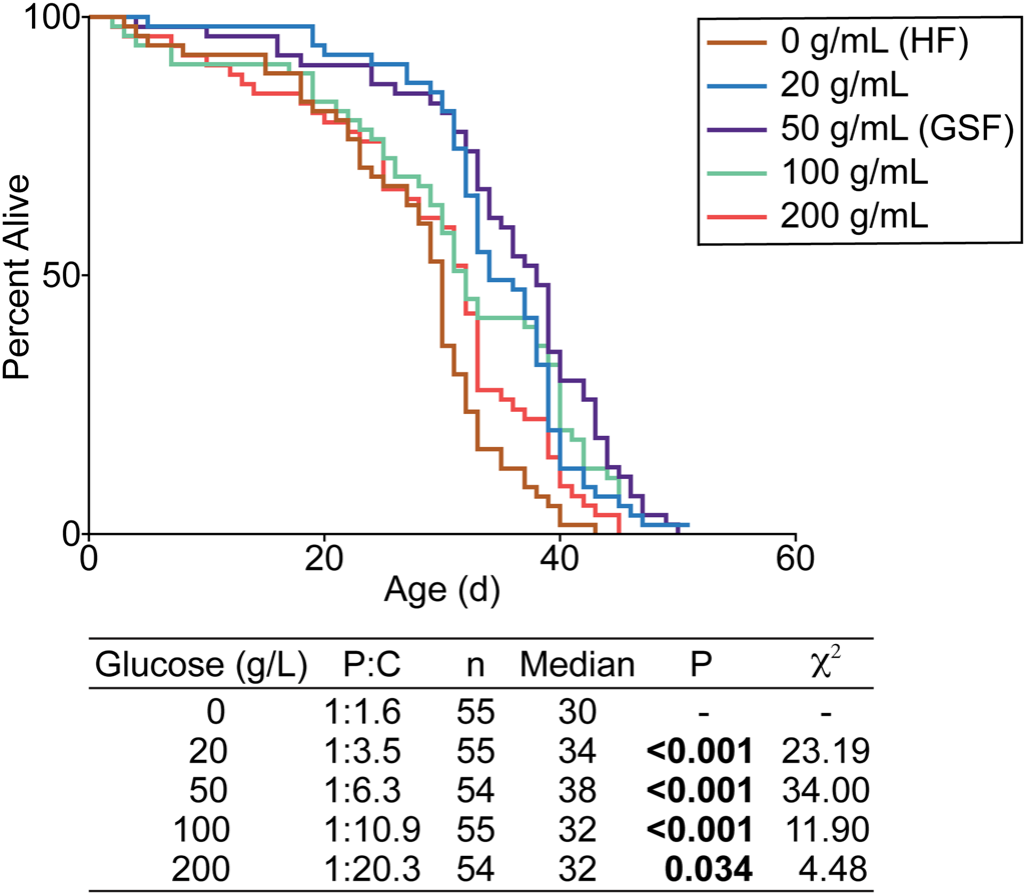
**Longevity of flies raised on holidic food supplemented with increasing glucose amounts.** Survival curve of *w^1118^* flies (*n*=54–55) raised on HF supplemented with glucose ranging from 0 ***g***/L to 200 ***g***/L. Experiment performed with single replicate. Results of log-rank (Mantel–Cox) test of data shown in table.

In a longitudinal study of relationships between nutrition, age, and metabolism, we found that, regardless of genetic background, glucose-supplemented (100 ***g***/L) holidic food extends the lifespan of adult *Drosophila* compared to unmodified holidic food, particularly in males (Galenza et al., 2016). As prolonged consumption of sugar-rich food is typically associated with diminished health and lifespan outcomes, we asked how addition of glucose extends longevity. Before addressing this question, we first tested a range of glucose concentrations to identify the optimal amount required for increased longevity. Specifically, we measured longevity of flies raised on holidic food that we supplemented with 0 to 200 ***g***/L glucose. We found that addition of 50 ***g***/L glucose had the greatest effect, leading to a 27% increase in median lifespan compared to unmodified food (Fig. 1). Thus, for the remainder of this study we determined the effects of holidic food (HF), and 50 ***g***/L glucose-supplemented holidic food (GSF) on health and longevity.

**Fig. 2.**
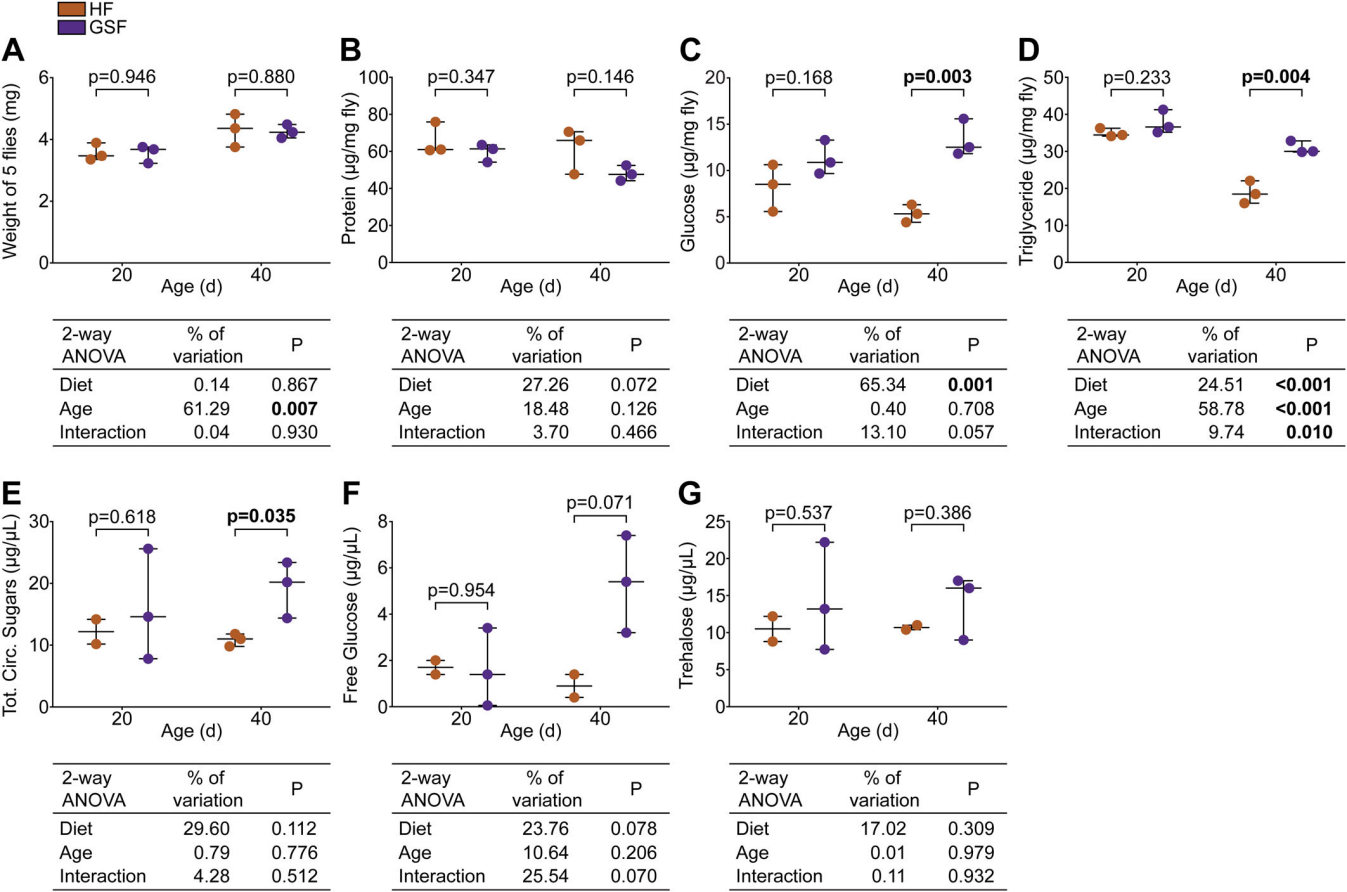
**Glucose-supplemented food promotes maintenance of macronutrients with age.** (A–D) Quantification of (A) weight, (B) protein, (C) glucose, and (D) triglycerides in *w^1118^* flies raised on GSF versus unmodified HF for 20 or 40 days (*n*=3). Each dot represents five flies. (E–G) Quantification of (E) total circulating sugars, (F) free glucose, and (G) trehalose in *w^1118^* flies raised on GSF or HF for 20 or 40 days (*n*=2–3). Statistical significance for (A–G) determined by two-way ANOVA. Further statistical analysis within age groups determined by Student's *t*-test (*P*<0.05).

We then quantified the impact of added glucose on metabolism by comparing weight and macronutrient content in wild-type flies raised on HF or GSF for 20 or 40 days. For each measurement, we performed a two-way ANOVA to analyze the contributions of age and diet to any detected changes. We found no differences in weights between diets at either at day 20 or 40, and weight increased significantly with age on both diets (Fig. 2A). Age did not appear to impact protein levels, and protein levels were not affected by GSF-treatment at either age, though they trended lower in flies raised on GSF compared to HF (Fig. 2B). In contrast, we found that diet significantly affected glucose level, as 40-day-old flies raised on GSF had higher glucose than those raised on HF (Fig. 2C). Age and interactions between age and diet also affected changes in triglycerides (Fig. 2D). For both diets, triglyceride levels declined with age, although the decrease was more pronounced in flies raised on HF than GSF, and GSF-fed flies had significantly more triglyceride by day 40. The effects of GSF-treatment on macronutrients reported here largely align with our previous observations using a higher sample size (*n*=5), where we observed similar effects on weight and glucose levels, though subtle differences exist for protein and triglycerides (Davoodi et al., 2019). In our previous study, we observed that protein levels were reduced significantly with both GSF-treatment and age, and age-dependent triglyceride decreases were more pronounced in flies raised on HF. Despite these differences, our data collectively show that GSF-treatment enhances maintenance of energy stores in older flies.

As GSF elevated total glucose content, we asked if GSF also impacted circulating glucose and trehalose, the primary blood sugar in insects. We found that diet had a mild effect on total circulating sugars, in older flies (Fig. 2E). Focussing on component circulating sugars, this difference is likely attributable to increased free glucose (Fig. 2F), with no detectable effects on trehalose (Fig. 2G). Combined, our data suggest that increased GSF-treatment contributes to the maintenance of energy-rich triglycerides and sugars, particularly as flies age.

### Glucose-supplemented food increases calorie intake

As our flies are fed *ad libitum*, we do not know if GSF-dependent effects on macronutrients are the indirect result of changes in feeding. We consider this an important question to address, as calorie intake and feeding frequency have been associated with lifespan changes in several experimental organisms (Fontana and Partridge, 2015).

**Fig. 3.**
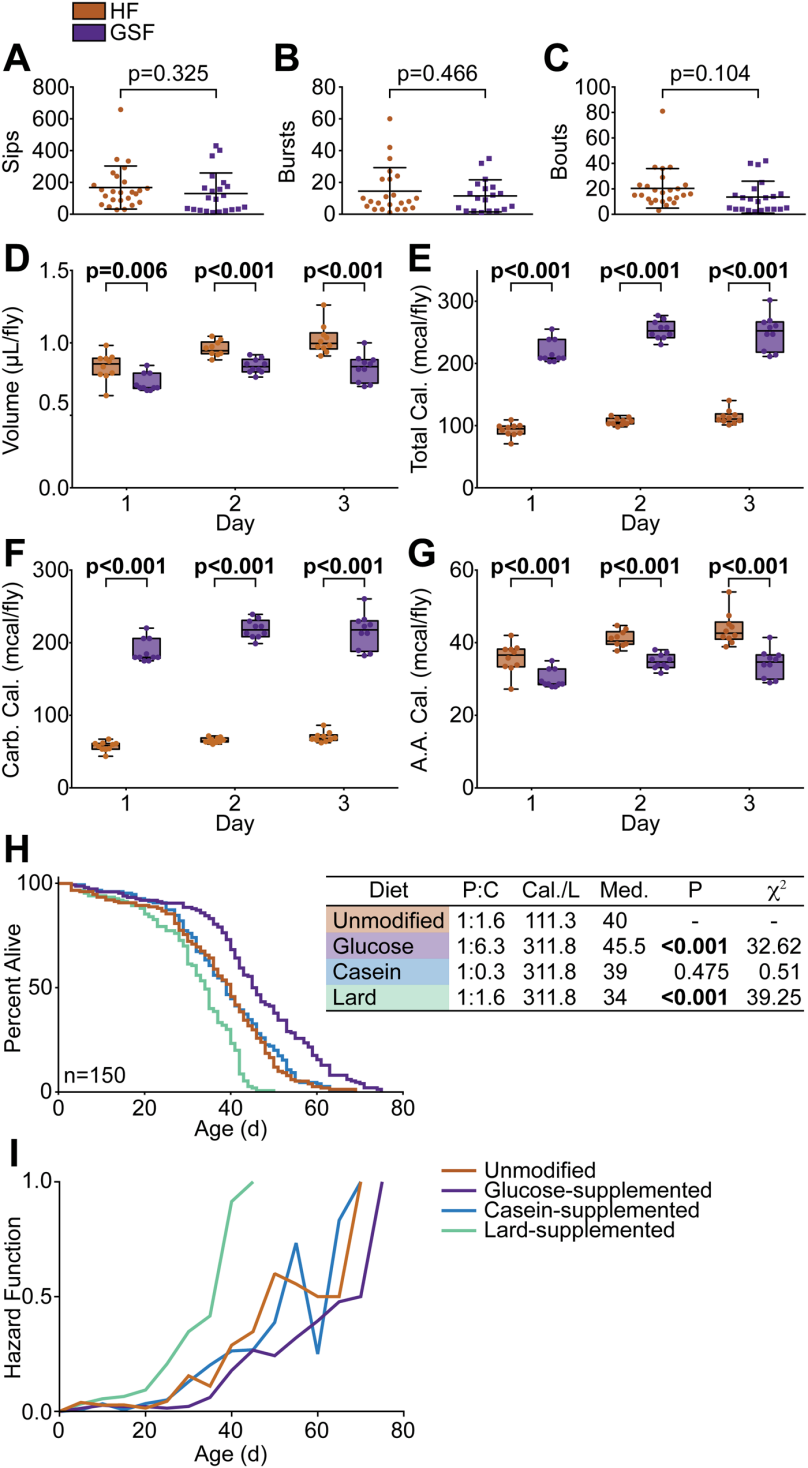
**Glucose-supplemented food increases caloric intake.** (A–C) Quantification of (A) number of sips, (B) feeding bursts, and (C) feeding bouts in 20-day-old *w^1118^* flies raised on GSF or unmodified HF using a flyPAD (*n*=32). (D–G) Quantification of liquid food consumption in 20-day-old *w^1118^* flies raised on GSF versus HF using a CAFE (*n*=10) measuring (D) volume consumed, (E) total calories, (F) calories from carbohydrates, and (G) calories from amino acids (AA). (H–I) (H) Survival curve and (I) hazard function of *w^1118^* flies raised on HF, GSF, casein-supplemented food, or lard-supplemented food (*n*=150). Longevity performed with single replicate. (H) Statistical significance determined by log-rank (Mantel–Cox) test shown in table.

To measure feeding frequency, we used the flyPAD (Itskov et al., 2014) to count individual sips; bursts, which are clusters of sips; and bouts, which are clusters of bursts, in flies raised on HF or GSF. For this assay, we raised flies on their respective foods for 20 days, then starved them for 2 h prior to feeding in a flyPAD arena for 1 h. We saw no difference in sips (Fig. 3A), bursts (Fig. 3B), or bouts (Fig. 3C), between flies raised on HF or GSF, suggesting that GSF does not significantly alter feeding behavior over short periods. However, it is worth considering that food contact may not correlate with consumption. To address this and determine if GSF impacts feeding behavior over longer timeframes, we used the capillary feeding (CAFE) assay (Ja et al., 2007), to calculate food consumption across three days. In the CAFE assay, flies are fed through capillary tubes that allow us to quantify liquid food consumption. We raised flies on HF or GSF for 20 days before transfer to the CAFE setup for a 3 day period, where flies were fed a liquid version of their respective food. We found that flies raised on HF consumed a greater volume than those raised on GSF, about a 1.2-fold daily increase (Fig. 3D). Accounting for macronutrient composition, this translates to a 2.3-fold increase in calorie intake for GSF-treated flies compared to HF-treated (Fig. 3E). The increased calorie intake is a result of elevated carbohydrate consumption, as flies raised on GSF consumed approximately 3.2-fold more calories from carbohydrates per day than their counterparts raised on HF (Fig. 3F). Conversely, amino acids provided approximately 20% fewer calories to flies raised on GSF than on HF (Fig. 3G). We note that both the flyPAD and CAFE assays are performed in different conditions than those used for the lifespan studies, so it is worth consideration that these data may not reflect the standard lab conditions used in this study. Nonetheless, our data show that flies raised on GSF consume significantly more calories in the form of carbohydrate, and fewer in the form of protein than flies raised on HF.

To test if the lifespan extension observed for flies raised on GSF is simply a consequence of feeding adults a higher calorie food, we measured lifespans of flies raised on modified holidic food isocaloric to GSF, where extra energy was provided either from lard, or casein. As expected, flies raised on GSF lived significantly longer than counterparts on HF (Fig. 3H). In contrast, casein-supplemented holidic food had no detectable effects on lifespan, whereas lard-supplemented holidic food shortened lifespan (Fig. 3H), and significantly increased the risk of early death (Fig. 3I). As increased levels of protein often decrease lifespan (Lee et al., 2008; Simpson and Raubenheimer, 2009), it was unexpected that the supplementation with casein had no effect. It is possible that the increased calories from casein offsets the effect of increased protein to carbohydrate ratio on lifespan, although further studies are required to test this hypothesis. However, as lard supplementation decreased lifespan, and protein supplementation had no effect, we conclude that simply adding extra calories to HF is not sufficient to extend longevity, indicating that GSF extends lifespan through a more specific mechanism.

### Glucose-supplemented food extends lifespan independent of insulin activity

As we observed increased total and circulating glucose in flies that we raised on GSF, we wondered what effects GSF has on the insulin pathway, a known modifier of longevity (Clancy et al., 2001; Tatar et al., 2003).

**Fig. 4.**
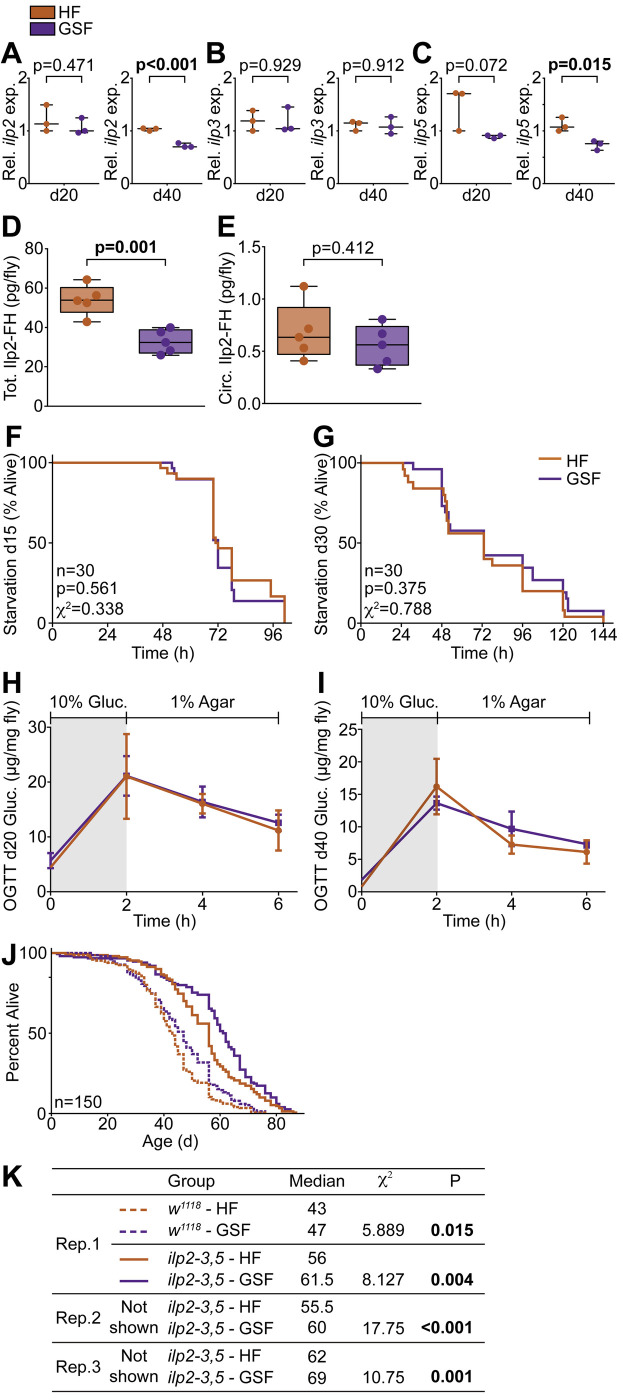
**Glucose-supplemented food extends lifespan independent of systemic insulin activity.** (A–C) Quantification of the relative expression of (A) *ilp2*, (B) *ilp3*, and (C) *ilp5* in *w^1118^* flies raised on GSF versus unmodified HF for 20 or 40 days (*n*=3). (D–E) Quantification of (D) total and (E) circulating Ilp2-FH in *w^1118^* flies raised on GSF versus HF for 20 days (*n*=5). Statistical significance (denoted by asterisk) for (A–E) determined by Student's *t*-test (*P*<0.05). (F–G) Survival curve upon starvation of *w^1118^* flies raised on GSF versus HF for (F) 15 or (G) 30 days (*n*=30). (H,I) Oral glucose tolerance test performed on *w^1118^* flies raised on GSF versus HF for (H) 20 or (I) 40 days (*n*=5). (J,K) Survival curve of *ilp2-3,5* flies raised on GSF versus HF (*n*=150). Longevity assay performed with three replicates, only one shown. Statistical significance for survival curves determined by log-rank (Mantel–Cox) test.

To answer this question, we quantified transcription of the insulin-like peptides (Ilp) *ilp2*, *ilp3*, and *ilp5*, in flies raised on HF or GSF for 20 or 40 days. Expression of *ilp2* and *ilp5* was lower in 40-day-old flies raised on GSF compared to flies raised on HF (Fig. 4A,C), while expression of *ilp3* was unaffected (Fig. 4B). In flies, *ilp* gene expression is complex, and does not necessarily reflect amounts of peptide in storage, or circulation (Park et al., 2014). Therefore, we used an ELISA to quantify total, and circulating amounts of FLAG and HA epitope-tagged Ilp2 (Ilp2-FH) in flies raised on GSF or HF. In this line, Ilp2-FH expression is controlled by the *ilp2* promoter, and accurately reports Ilp2 peptide levels (Park et al., 2014). We observed significantly lower total amounts of Ilp2-FH in GSF-treated flies compared to age-matched HF-treated controls (Fig. 4D). However, we did not detect food-specific effects on levels of circulating Ilp2-FH (Fig. 4E).

To determine whether GSF-dependent shifts in insulin peptide expression translate into effects on insulin activity, we measured starvation resistance and oral glucose tolerance in flies raised on GSF and HF. In flies, insulin impairs starvation resistance, and improves glucose tolerance. Thus, we expect that any effects of GSF on insulin signaling will have measurable impacts on starvation resistance or glucose tolerance. For starvation assays, we raised flies on HF, or GSF, for 15 or 30 days, and followed survival after switching to nutrient-deficient medium. For both ages, we did not detect food-dependent effects on starvation resistance (Fig. 4F,G). Interestingly, increased triglyceride levels, as observed in GSF-fed flies, typically corresponds with increased starvation resistance (Heier and Kühnlein, 2018), though we did not observe that here.

For the oral glucose tolerance test (OGTT) we raised flies on HF or GSF for 20 or 40 days, followed by a 16 h fast, prior to a 2 h *ad libitum* feed on a 10% glucose medium, followed by re-fasting. We quantified total glucose in flies following the initial fast (0 h), after feeding on 10% glucose (2 h), and twice during the re-fast period (4 h, 6 h). In insulin-sensitive flies, glucose levels rise during feeding, and drop during the fast, due to insulin-dependent stimulation of glucose uptake. We found that flies raised on either food processed glucose with equal efficiency at all time points in both ages (Fig. 4H,I), arguing that GSF does not significantly impair insulin sensitivity as flies age.

Finally, we measured the lifespans of HF and GSF-treated *ilp2-3*, *5* mutant flies. *ilp2-3*, *5* mutants are deficient for systemic insulin signaling, and normally outlive wild-type controls. Thus, if GSF extends lifespan by suppressing systemic insulin activity, we expect that *ilp2-3*, *5* mutants will not benefit from lifelong culture on GSF. As expected, *w^1118^* controls raised on GSF outlived those raised on HF, though not to the same extent as is Fig. 1, likely reflecting inherent variability in lifespan assays. Contrary to our hypothesis, *ilp2-3*, *5* mutants raised on GSF significantly outlived *ilp2-3*, *5* mutants raised on HF (Fig. 4J, K), a phenotype we replicated in three independent assays (Fig. 4K). Thus, although GSF has effects on the expression of two insulin peptide genes, we did not detect GSF-dependent effects on insulin activity, or survival of insulin-deficient flies. As we did not observe a sign of functional insulin defects, we believe our data are most consistent with a hypothesis that that GSF extends life through insulin-independent means.

### Glucose-supplemented food increases expression of intestine-associated cell–cell junction genes

**Fig. 5.**
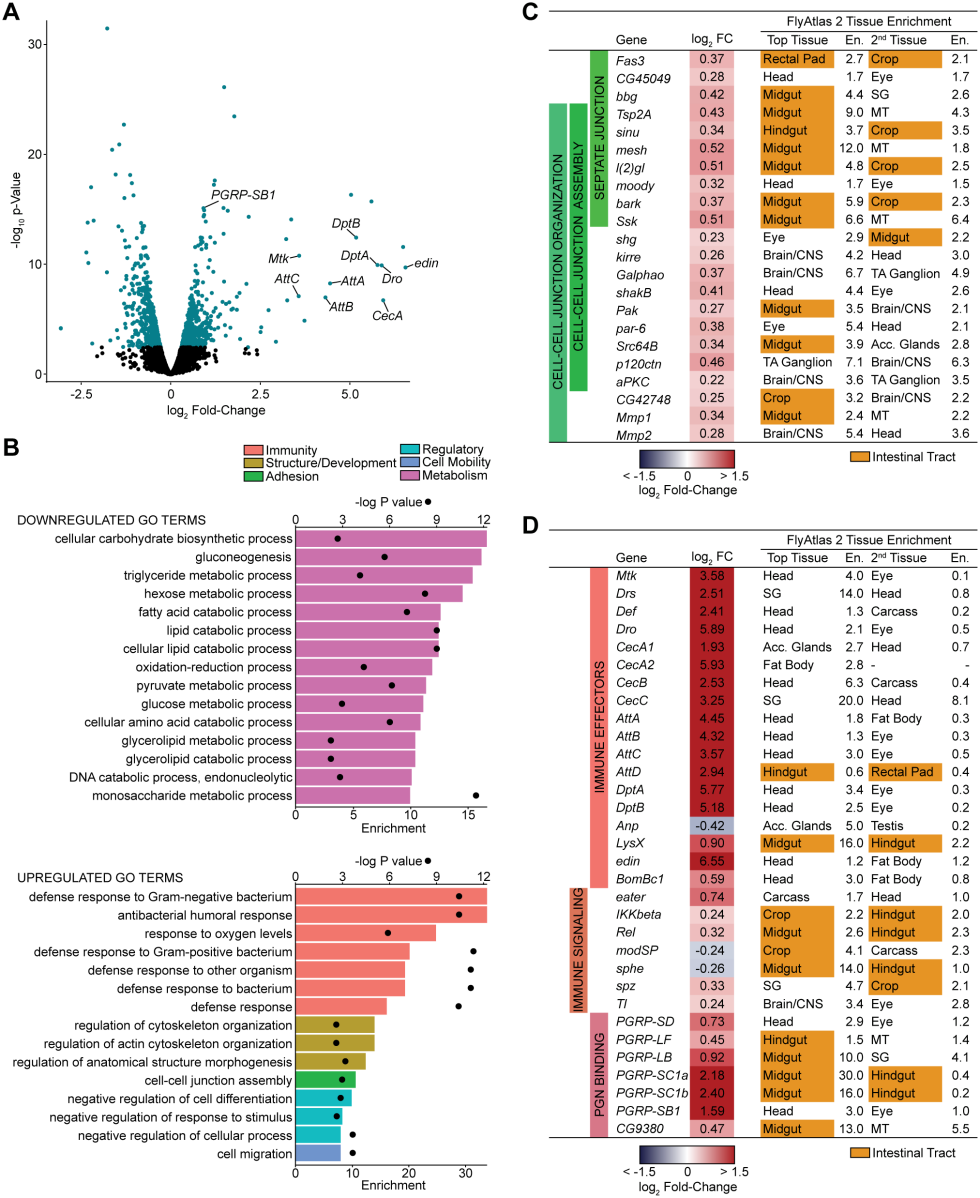
**Glucose-supplemented food increases expression of intestine-associated cell–cell junction genes.** (A) Volcano plot of differentially expressed genes from comparison of flies raised on GSF versus unmodified HF. Each dot represents a single gene. Teal indicates *P*<0.01, FDR <0.05. (B) Gene Ontology (GO) analysis from down- or upregulated differentially expressed genes from comparison of flies raised on GSF versus HF. Bars (bottom x-axis) represent enrichment scores and black circles (top x-axis) represent -logP values for each enriched GO term. (C–D) Differentially expressed (*P*<0.05) (C) cell junction genes or (D) immune-related genes from comparison of flies raised on GSF versus HF. Tissue enrichment is shown for tissues with the first and second highest enrichment scores based on FlyAtlas2 output of these genes.

To determine how GSF extends longevity, we used RNA sequencing (RNA-Seq) to compare transcription in whole flies raised on GSF or HF for 20 days. When we looked at differential gene expression, we found 488 upregulated genes and 555 downregulated genes in GSF-fed flies compared to HF-fed controls (Fig. 5A). Gene ontology analysis of downregulated processes showed that GSF primarily leads to diminished expression of genes required for metabolism, and energy use (Fig. 5B). In particular, we noticed significant decreases in expression of genes involved in gluconeogenesis and lipid catabolism (Fig. 5B), likely a result of the increased availability of glucose as an energy source, and consistent with our observation that flies raised on GSF have elevated triglycerides relative to HF-treated counterparts (Fig. 2D).

In contrast to the dominance of metabolic terms among downregulated gene ontologies, we found that GSF enhanced expression of genes involved in a number of distinct cellular processes, including immunity, cell adhesion, and cell mobility (Fig. 5A,B). In fact, many of the genes with the highest GSF-dependent changes in gene expression encode antimicrobial peptides such as *attacins* and *diptericins* (Fig. 5A,D). While upregulation of immune gene expression may appear unexpected, we observed a similar phenomenon in microarray analysis comparing 10-day-old female flies raised on 100 ***g***/L glucose-supplemented HF to females raised on unmodified HF (Fig. S1). Within the list of enriched gene ontology terms for upregulated genes, we were struck by increased expression of genes associated with cell-cell junctions (Fig. 5B,C). Cell–cell junctions are critical for maintenance of epithelial structures, particularly in the intestinal tract, where epithelial barrier damage is linked to mortality (Rera et al., 2012). When we used the online resource FlyAtlas 2 to identify tissues that prominently express GSF-responsive cell–cell junction genes, we noted that a substantial number are highly expressed in the intestinal tract (Fig. 5C). To confirm this, we compared transcription of representative cell–cell junction genes in whole flies, dissected heads as a control tissue, and dissected intestines. For all genes, we noted enriched expression in the intestinal tract relative to whole flies, or dissected heads (Fig. S2), raising the possibility that GSF impacts organization of the gut epithelial barrier.

### Glucose-supplemented food improves intestinal barrier integrity

Intestinal barrier integrity deteriorates with age and a weakened barrier is associated with reduced lifespan (Rera et al., 2012). As we observed increased expression of cell–cell junction genes in GSF-treated flies, we asked what effects GSF has on barrier integrity.

**Fig. 6.**
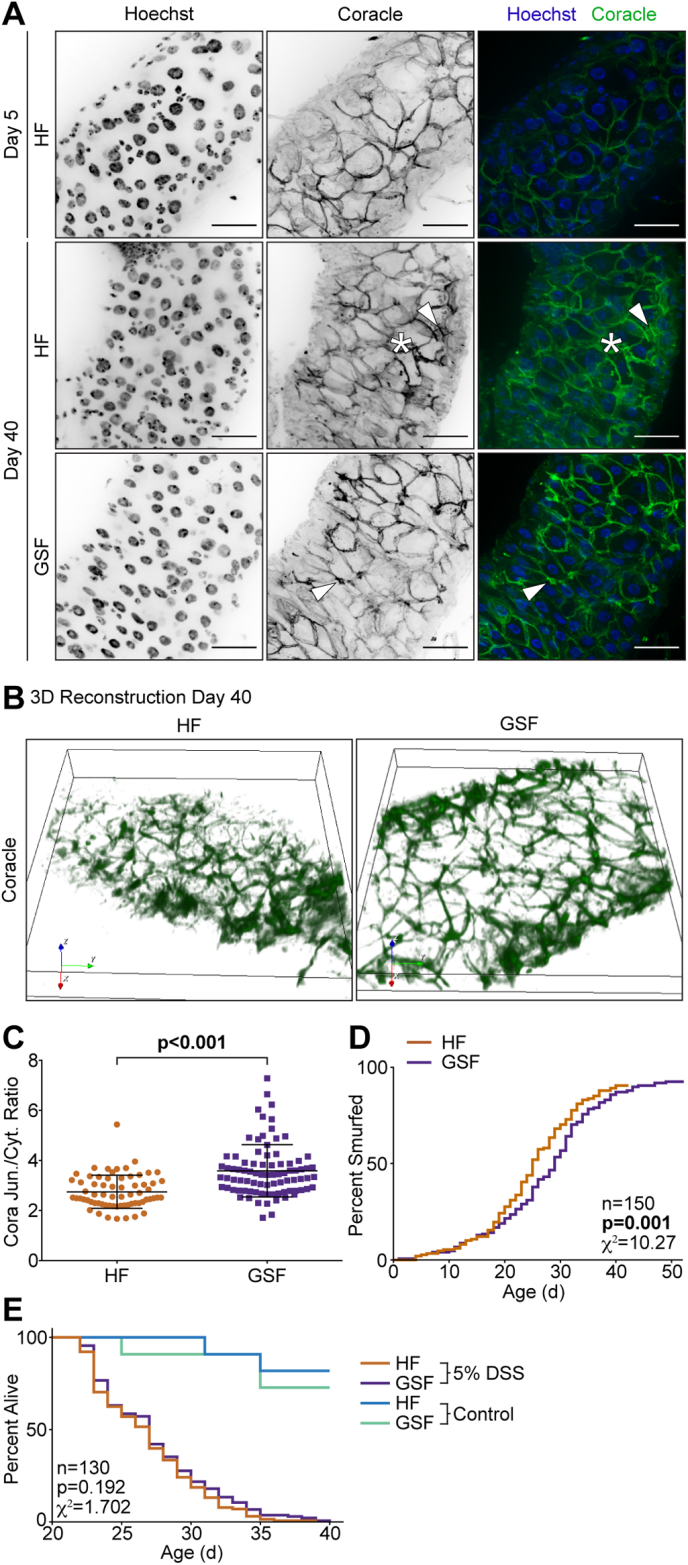
**Glucose-supplemented food improves intestinal barrier integrity.** (A) Immunofluorescent images of the posterior midgut of 5- or 40-day-old *w^1118^* flies raised on unmodified HF or GSF labeling DNA (Hoechst, blue) and Coracle (green). Scale bars, 25 µm. (B) 3D reconstruction images of Coracle in the posterior midgut of 40-day-old *w^1118^* flies raised on HF or GSF. (C) Quantification of Coracle as a ratio of junction to cytoplasm localization in the posterior midgut of 40-day-old *w^1118^* flies raised on HF (*n*=7 guts, 66 cells) or GSF (*n*=8 guts, 84 cells). (D) Measurement of smurfs over time in *w^1118^* flies raised on HF or GSF (*n*=150). (E–F) (E) Experimental design and (F) survival curves of *w^1118^* flies raised on HF or GSF for 20 days, then transferred to food supplemented with 5% dextran sodium sulfate (DSS) (*n*=130) or control food (*n*=10). Survival experiment performed with single replicate. Statistical significance for survival curves determined by log-rank (Mantel–Cox) test.

The fly gut epithelial barrier is maintained by septate junctions, which are analogous to mammalian tight junctions. Coracle (Cora), a *Drosophila* protein 4.1 homolog, is an essential component of septate junctions. As flies age, Cora and other septate junction proteins partially lose their cell junction localization and accumulate in the cytosol, leading to breaches in the barrier, paracellular leak of lumenal material into interstitial tissue, and ultimately, death (Rera et al., 2012; Resnik-Docampo et al., 2017). To determine effects of GSF on the intestinal barrier, we used immunofluorescence to examine the cellular distribution of Cora in intestines of 40-day-old flies raised on HF or GSF compared to 5-day-old flies raised on HF. The intestines of 5-day-old flies raised on HF contained orderly arrangements of large, polyploid nuclei of absorptive enterocytes, and smaller, evenly spaced nuclei of progenitor cells or secretory enteroendocrine cells (Fig. 6A, Hoechst). At this young age, septate junctions are easily identified as fine margins of Cora staining (Fig. 6A, Coracle). In 40-day-old flies raised on HF, we noted classic hallmarks of age-dependent epithelial degeneration. Specifically, we detected unevenly distributed, large enterocyte nuclei, interspersed by irregular populations of smaller nuclei from progenitor/enteroendocrine cells (Fig. 6A, Hoechst). In addition, we detected cytosolic accumulations of Cora (Fig. 6A, asterisk), including enrichment in punctae (Fig. 6A, arrowhead). In contrast, age-matched intestines of flies raised on GSF looked more similar to younger flies raised on HF, with regularly spaced nuclei (Fig. 6A, Hoechst), while Cora distribution appeared more localized to junctions than in HF-fed samples (Fig. 6A, Coracle). 3D reconstruction of 40-day-old intestines highlighted the difference in Cora localization between flies raised on HF or GSF (Fig. 6B). In flies raised on GSF, Cora retained a reticulated pattern associated with points of cell–cell contact at septate junctions. In contrast, we detected uneven, diffuse Cora distribution in intestines from age-matched flies raised on HF.

To quantify food-dependent impacts on subcellular distribution of Cora, we determined the junction to cytosol ratio of Cora in midguts of flies raised on HF or GSF for 40 days. Here, we detected significantly higher junction to cytosol ratios of Cora in 40-day-old GSF-treated flies than in age-matched HF-treated flies (Fig. 6C), supporting the hypothesis that GSF sustains Cora association with septate junctions as flies age.

To test if GSF functionally improves barrier integrity in aged flies, we performed a smurf assay, in which a non-permeable dye, that only crosses the epithelium upon loss of barrier integrity, is added to the food. Flies raised on GSF smurfed significantly later than those on HF (Fig. 6D), confirming enhanced barrier integrity in GSF-treated flies. Finally, we asked if disrupting the epithelial barrier reverts the lifespan benefits associated with GSF. For this experiment, we raised flies on GSF or HF for 20 days, at which point we transferred them to HF or GSF that we supplemented with 5% dextran sodium sulfate (DSS), a detergent that disrupts the gut barrier (Fig. 6E). By increasing intestinal permeability with DSS, we found that flies raised on GSF completely lost their survival advantage (Fig. 6F), perishing at the same time as flies raised on HF, suggesting that GSF-dependent lifespan requires an intact intestinal epithelial barrier.

## DISCUSSION

Aging and age-related diseases pose a growing global challenge. Dietary interventions offer a promising approach to improve aging, but questions remain regarding optimal regimes. Here, we asked how glucose-supplemented food (GSF) extends adult male *Drosophila* longevity. Our data suggest that GSF-dependent lifespan extension is not an effect of lower caloric intake, or systemic insulin activity, two frequently studied regimes of lifespan extension. Instead, we found that flies raised on GSF increased expression of cell junction genes, and had an extended duration of gut barrier function. Furthermore, our work showed that chemical disruption of the intestinal barrier removed the lifespan extension associated with GSF-treatment. Thus, our data are consistent with a hypothesis that GSF prolongs adult viability by maintaining intestinal barrier integrity, although detailed mechanistic studies are required to test this hypothesis.

While we found that glucose supplementation extends lifespan in flies, in contrast, studies in other organisms have found differing effects from glucose. In particular, recent studies using the *Caenorhabditis elegans* model have found mixed outcomes on lifespan from glucose supplementation. Providing *C. elegans* 5–50 mM glucose shortens lifespan (Schlotterer et al., 2009; Schulz et al., 2007). Interestingly, high glucose (2% or 111 mM) treatment in young worms (1–3 days old) reduces lifespan, but beginning glucose treatment after worms are at a post-reproductive age (7 days old) extends lifespan (Lei et al., 2018 preprint). A recent study found a direct effect of glucose metabolism on aging in worms, where glycolysis is detrimental to, and gluconeogenesis is beneficial to healthy aging, though not maximal longevity (Onken et al., 2020). As glucose metabolism is evolutionarily conserved, it will be of interest to explore the role of glycolysis and gluconeogenesis in GSF-dependent lifespan extension in flies and vertebrate models. Likewise, it will be of value to ask if glucose affects intestinal barrier integrity in other models, as we observe in flies.

The epithelial barrier is essential for health and longevity. Occluding junctions, known as tight junctions in vertebrates, or the related septate junctions of invertebrates, allow regulated movement of extracellular material across the epithelium (Zihni et al., 2016). Disrupted expression and localization of tight junction components are observed in Crohn's disease (Zeissig et al., 2007) and sepsis (Yoseph et al., 2016), with the upregulation of pore-forming claudin-2 and downregulation of sealing claudin-5 in both cases. Intestinal permeability also increases with age, as occluding junction proteins are downregulated (Parrish, 2017). In flies, formation and maintenance of septate junction protein complexes relies on several proteins including Mesh (Izumi et al., 2012), Snakeskin (Yanagihashi et al., 2012), and Coracle (Lamb et al., 1998). Similar to vertebrates, disruption of septate junctions affects intestinal health and longevity. For example, loss of Snakeskin alters composition of the gut bacterial community, and upregulation of Snakeskin extends lifespan (Salazar et al., 2018).

Effects of GSF on the intestinal barrier are consistent with literature that linked food intake to intestinal permeability, frequently by targeting occluding junctions (De Santis et al., 2015). For example, the amino acid glutamine has received interest for its therapeutic potential in intestinal health, as glutamine directly and indirectly upregulates tight junction protein levels (Wang et al., 2015). Conversely, gliadin, a component of wheat, increases intestinal permeability in celiac disease by disassembly of tight junctions (Schumann et al., 2017). Gliadin binds CXCR3, inducing a MyD88-dependent release of zonulin. Loss of zonulin weakens tight junctions by altering the localization of junction proteins (Lammers et al., 2008). With their analogous role and many conserved proteins, studying the septate junctions of *Drosophila* will provide a useful *in vivo* model to explore relationships between food and the integrity of occluding junctions.

Although we did not identify the molecular mechanism by which GSF improves intestinal barrier integrity, others have explored the effect of glucose on epithelial barriers. Exposure of human retinal pigment epithelial cells to high glucose (25 mM compared to 5.5 mM) improved barrier function by increased expression of tight junction proteins (Villarroel et al., 2009). Conversely, hyperglycemia in mice, induced by streptozotocin treatment, drives intestinal barrier dysfunction by global transcriptional reprogramming of intestinal epithelial cells, including downregulation of N-glycan biosynthesis genes (Thaiss et al., 2018), a critical pathway for tight junction assembly (Nita-Lazar et al., 2010). Remarkably, treatment with insulin restored the intestinal barrier function of streptozotocin-treated mice, suggesting insulin regulation of barrier integrity through tight junctions. Our data support a mechanism independent of systemic insulin activity, but it is worth consideration in future studies that intestinal insulin expression may regulate barrier integrity.

Changes to protein content of food have documented effects on longevity (Fontana and Partridge, 2015; Mair et al., 2005). Work with yeast, *C. elegans*, and *Drosophila* found that lowering protein levels may extend lifespan in part through reduction in Target of Rapamycin (TOR) activity (Fontana et al., 2010; Kapahi et al., 2010). Though our study was not designed to test interactions between protein and lifespan, our CAFE assay data indicate that flies raised on GSF received 14% of their calories from protein, whereas flies raised on HF received 38% of their calories from protein. A recent study in flies suggests that lowering dietary yeast levels, an effective decrease in protein levels, enhances barrier function via Myc activity in intestinal enterocytes (Akagi et al., 2018). Our RNA-seq data did not uncover differential expression of the *myc* gene in GSF-fed flies. However, we cannot exclude the possibility that GSF may improve barrier integrity in a Myc-dependent manner. Lipid metabolism has been increasingly linked to the aging process in several model organisms (Johnson and Stolzing, 2019), and our RNA-sequencing analysis showed that GSF treatment reduces numerous metabolic genes, including those involved in lipid metabolism. Unexpectedly, increased triglyceride levels in GSF-fed flies failed to protect them against starvation (Heier and Kühnlein, 2018), suggesting that GSF-fed flies may have reduced capacity for lipid mobilization. Collectively, these data emphasize the complexity of interactions between macronutrient availability and host longevity, and indicate the importance of considering effects of Myc, protein and triglyceride on the intestinal barrier.

While we focussed on cell-junction genes in this report, we also observed a striking increase in expression of immune-related genes, particularly antimicrobial peptides, in GSF-treated flies. This was unexpected, as antimicrobial peptide expression increases with age (Pletcher et al., 2002), and promotes intestinal barrier dysfunction (Rera et al., 2012). Selective breeding for long-lived flies reduces age-dependent increase in antimicrobial peptide expression (Fabian et al., 2018). Furthermore, knockdown of individual antimicrobial peptides extends lifespan (Lin et al., 2018). The effect of overexpression of antimicrobial peptides on lifespan may be context-dependent as evidence suggests either detrimental (Badinloo et al., 2018) or beneficial outcomes (Loch et al., 2017). Higher baseline antimicrobial peptide expression in the long-lived GSF-treated flies suggests that the relationship between antimicrobial peptides and lifespan may be complex. As we performed RNA-sequencing on 20-day-old flies, it would be of interest to measure antimicrobial peptide expression in GSF-treated flies across their lifespan to determine changes with age.

While our study suggests that supplementation of glucose to holidic food extends lifespan through enhanced intestinal barrier integrity, it is important to acknowledge that limitations in our study prevent us from establishing a causal mechanism. Disruption of the intestinal barrier through DSS-treatment removed the survival advantage of GSF-fed flies, supporting a role for barrier integrity in GSF-mediated lifespan extension. However, DSS treatment was also detrimental to flies raised on either diet compared to untreated controls. Thus, further studies of how GSF affects the intestinal barrier with interventions that are less harmful will help to provide this mechanistic insight. As we mainly compared two diets in this study, we cannot definitively state whether increased glucose intake, or if an alternative difference between the two diets, such as the protein to carbohydrate ratio or restriction of protein intake, leads to improved barrier integrity and lifespan extension. A thorough, comprehensive study in line with the Geometric Framework for Nutrition will be required to determine if glucose-supplemented food extends lifespan because of its lower protein to carbohydrate ratio relative to unmodified holidic food or reduced protein intake (Lee et al., 2008). Furthermore, while we examined the effect of glucose supplementation, it may be possible that other carbohydrates, such as sucrose or fructose, impact lifespan through a similar mechanism and future studies will be required to investigate this.

In this study, we performed experiments on virgin male flies, though we previously found that 100 ***g***/L glucose-supplemented food also improved lifespan in virgin females (Galenza et al., 2016). Recent reports have revealed distinct sex differences in intestinal physiology, including a higher proliferative rate in intestinal stem cells of females, that could affect the response to dietary interventions (Hudry et al., 2016; Millington and Rideout, 2018). In females, the high nutritional requirements of oogenesis may contribute to these distinct responses compared to males (Wu et al., 2020). The metabolic response to sugar itself is distinct between sexes, as bi-directional communication between male gonads and the proximal intestine drives a male-biased increased expression of sugar metabolism genes in the midgut (Hudry et al., 2019). As we focused on virgin males, it is worth consideration that the lifespan extension associated with glucose supplementation and observed physiological changes may be different in females or mated flies.

This study shows that moderate levels of glucose may extend *Drosophila* lifespan through improved intestinal barrier integrity. In humans, the intestinal barrier deteriorates with age, as well as in chronic diseases such as inflammatory bowel disease. With population aging becoming a growing global concern, further investigation of how dietary components can help maintain intestinal barrier integrity will be essential. We believe that these findings contribute to our understanding of intestinal health and may help efforts to develop preventative measures to limit the effects of aging and disease.

## MATERIALS AND METHODS

### *Drosophila* husbandry

Virgin male *w^1118^* flies were used for all experiments unless otherwise specified. Other fly lines used were *Df(3L)Ilp2-3*,*Ilp5^3^* and *ilp2^1^ gd2HF* (Park et al., 2014). Flies were maintained at 25°C on a 12 h light:12 h dark cycle and flipped to fresh food every 2–3 days. Flies in this study were allowed to develop on BDSC cornmeal food (https://bdsc.indiana.edu/information/recipes/bloomfood.html). Upon emergence, adults were transferred to their respective holidic food. The holidic food (HF) was prepared following the published protocol and recipe using the original amino acid solution (Oaa) at 100 mM biologically available nitrogen (Table S1) (Piper et al., 2014). Variants to this diet included supplementation with either 50 ***g***/L glucose (GSF), 50 ***g***/L casein, or 22.2 ***g***/L lard. For starvation assays, flies were maintained on 1% agar vials.

### Lifespan analysis

Virgin male flies were used for all lifespan studies. Lifespan studies were performed with 30 flies/vial. Flies were maintained at 25°C on a 12 h light:12 h dark cycle in a humidified incubator. Flies were flipped to fresh food every 2–3 days. Deaths were recorded daily.

### Macronutrient assays

Each assay was performed with three biological replicates, consisting of five flies per replicate. Each replicate was weighed and then mashed in 125 µL TET buffer (10 mM Tris, 1 mM EDTA, 0.1% Triton X-100, pH 7.4). Samples were centrifuged to remove cuticle debris. Macronutrient measurements were performed in 96-well plates using commercial kits: DC Protein Assay kit (Bio-Rad, 500-0116), Triglyceride Assay kit (Sigma-Aldrich, TG-5-RB), and Glucose (GO) Assay kit (Sigma-Aldrich, GAGO20). Colorimetric readings were obtained using a microplate spectrophotometer (Molecular Devices, SpectraMax M5).

To measure circulating sugars, each assay was performed with three biological replicates consisting of hemolymph drawn from 15–20 flies per replicate (Tennessen et al., 2014). Flies were carefully pierced in the thorax with a 26G needle and placed in a filter collection tube. Tubes were centrifuged at 9000 ***g*** for 5 min at 4°C yielding at least 1 µl of hemolymph. 1 µl of hemolymph was diluted 1:100 in trehalase buffer (5 mM Tris pH 6.6, 137 mM NaCl, 2.7 mM KCl), and placed in a 70°C water bath for 5 min. Each sample was split into two 50 µl aliquots, one to measure glucose and one to measure trehalose. Trehalase was prepared by diluting 3 µl porcine trehalase (1 UN) in 1 ml trehalase buffer. 50 µl of this trehalase solution was added to one aliquot of each sample while 50 µl trehalase buffer was added to the other, then samples were incubated at 37°C for 24 h. 30 µl of samples and standards were added to a 96-well plate and glucose was measured using the glucose oxidase (GO) assay kits (Sigma-Aldrich, GAGO20). Total circulating sugars was measured from the trehalase-treated sample, free glucose was measured from the untreated sample, and trehalose was calculated as the difference between treated and untreated samples.

### Enzyme-linked immunosorbent assay (ELISA)

To measure total and circulating Ilp2 levels, the *ilp2^1^ gd2HF* fly stock and protocols were provided by Dr Seung K. Kim (Park et al., 2014). Note that a different published protocol for hemolymph extraction was used compared to circulating sugar measurement. Each assay was performed with five biological replicates. To prepare each replicate, the black posterior was removed from ten males, and the remaining bodies were transferred to 60 µl PBS, followed by a 10 min vortex at maximum speed. Tubes were centrifuged at 1000 ***g*** for 1 min, then 50 µl of the supernatant was transferred to a PCR tube as the circulating Ilp2-FH sample. To the tubes with the remaining flies, 500 µl of PBS with 1% Triton X-100 was added, homogenized with a pestle and cordless motor (VWR 47747-370), and followed by a 5 min vortex at maximum speed. These tubes were centrifuged at maximum speed for 5 min, then 50 µL of the supernatant was transferred to a PCR tube, as the total Ilp2-FH sample.

For the ELISA, we used FLAG(GS)HA peptide standards (DYKDDDDKGGGGSYPYDVPDYA amide, 2412 Da: LifeTein LLC). 1 µl of the stock peptide standards (0–10 ng/ml) was added to 50 µl PBS or PBS with 1% Triton X-100. Wells of a Nunc Maxisorp plate (Thermo Scientific 44-2404-21) were coated with 100 µl of anti-FLAG antibody diluted in 0.2 M sodium carbonate/bicarbonate buffer (pH 9.4) to 2.5 µg/ml, then the plate was incubated at 4°C overnight. The plate was washed twice with PBS with 0.2% Tween 20, then blocked with 350 µl of 2% bovine serum albumin in PBS at 4°C overnight. Anti-HA-Peroxidase, High Affinity (clone 3F10) (Roche 12013819001, 25 µg/ml) was diluted in PBS with 2% Tween at a 1:500 dilution. 5 µl of the diluted anti-HA-peroxidase was added to the PCR tubes containing 50 µl of either samples or standards, vortexed, and centrifuged briefly. Following blocking, the plate was washed three times with PBS with 0.2% Tween 20. Samples and standards were transferred to wells of the plate, the plate was sealed with adhesive sealer (BIO-RAD, MSB-1001), and then placed in a humid chamber at 4°C overnight. Samples were removed with an aspirator and the plate was washed with PBS with 0.2% Tween 20 six times. 100 µl 1-Step Ultra TMB – ELISA Substrate (ThermoFisher Scientific 34028) was added to each well and incubated at room temperature for 30 min. The reaction was stopped by adding 100 µl 2 M sulfuric acid and absorbance was measured at 450 nm on a Spectramax M5 (Molecular Devices).

### Consumption assays

Both the flyPAD and the CAFE assays were used to characterize consumption in this study. The fly Proboscis and Activity Detector (flyPAD) records changes in capacitance to detect physical interaction of an individual fly with their food (Itskov et al., 2014). For the flyPAD assay, flies were starved for 2 h prior to the assay. HF and GSF was prepared as described, but with agarose substituted for the agar. Prepared food was melted at 95°C and then maintained at 60°C to facilitate pouring. Individual flies were placed in each flyPAD arena using a mouth aspirator at *n*=32 for each sample. Eating behaviour was recorded for 1 h.

The Capillary Feeder (CAFE) assay allows quantification of ingested liquid food over an extended period (Ja et al., 2007). For the CAFE assay, flies were maintained in empty vials at ten flies per vial with ten vials per sample (*n*=10) and fed liquid food through capillary tubes. To prepare liquid food for this assay, HF and GSF were prepared as described, but without the addition of agar. Flies were fed the liquid version of their respective diets for a period of 3 days. Food consumption was measured every 24 h, and fresh food was provided each day.

### Oral glucose tolerance test (OGTT)

Glucose tolerance was measured using an OGTT (Palu and Thummel, 2016). Each assay was performed with five biological replicates consisting of five flies per replicate. Flies were starved overnight for 16 h on 1% agar, switched to vials containing 10% glucose and 1% agar for 2 h, and then re-starved on vials of 1% agar. Samples were obtained after initial starvation, after 2 h on 10% glucose, and then at both 2 h and 4 h following re-starvation. Samples of five flies were weighed and then mashed in 125 µl TET buffer (10 mM Tris, 1 mM EDTA, 0.1% Triton X-100, pH 7.4). Glucose was measured using the GO assay kits (Sigma-Aldrich, GAGO20).

### RNA isolation and RT-qPCR

To isolate RNA for both RT-qPCR and RNA-seq, samples of five whole flies (or ten dissected heads, thoraxes, or intestines where specified) were homogenized in 250 µl TRIzol, then incubated at room temperature for 5 min. Samples were centrifuged at 12000 ***g*** for 10 min at 4°C. Clear homogenate was transferred to a 1.5 ml Eppendorf tube, then 50 µl of chloroform was added, shaken vigorously for 15 s, and incubated at room temperature for 3 min. Samples were centrifuged at 12,000 ***g*** for 15 min at 4°C. The upper aqueous layer was transferred to a 1.5 ml Eppendorf tube, 125 µl isopropanol was added, then left at −20°C overnight. Samples were centrifuged at 12,000 ***g*** for 10 min at 4°C. The RNA pellet was washed with 500 µl 75% ethanol, centrifuged at 7500 ***g*** for 5 min at 4°C, then allowed to air dry. The RNA pellet was dissolved in RNAse free water, then incubated at 37°C for 30 min with 1 µl DNAse.

For RT-qPCR, the following primers were used in this study: *ilp2* [forward (F): 5′-TCC ACA GTG AAG TTG GCC C-3′, reverse (R): 5′-AGA TAA TCG CGT CGA CCA GG-3′], *ilp3* (F: 5′-AGA GAA CTT TGG ACC CCG TGA A-3′, R: 5′-TGA ACC GAA CTA TCA CTC AAC AGT CT-3′), *ilp5* (F: 5′-GAG GCA CCT TGG GCC TAT TC-3′, R: 5′-CAT GTG GTG AGA TTC GGA GCT A-3′), and *rp49* (F: 5′- AAG AAG CGC ACC AAG CAC TTC ATC-3′, R: 5′-TCT GTT GTC GAT ACC CTT GGG CTT-3′). All RT-qPCR studies were performed with three biological replicates per sample (*n*=3), and relative expression values were calculated using delta–delta Ct calculations. Expression levels were normalized to *rp49*.

### RNA-sequencing analysis

An average of 60 million reads were obtained per biological replicate. Quality check was performed with FastQC to evaluate the quality of raw, paired-end reads. Adaptors and reads of less than 36 base pairs in length were trimmed from the raw reads using Trimmomatic (version 0.36). HISAT2 (version 2.1.0) was used to align reads to the *Drosophila* transcriptome-bdgp6, and the resulting BAM files were converted to SAM files using SAMtools (version 1.8). Converted files were counted with Rsubread (version 1.24.2) and loaded into EdgeR. In EdgeR, genes with counts less than one count per million were filtered and libraries normalized for size. Normalized libraries were used to identify genes that were differentially expressed between treatments. Genes with *P* value <0.01 and FDR <0.05 were defined as differentially expressed genes. Panther was used to determine GO term enrichment of downregulated and upregulated gene sets. FlyAtlas2 was used for tissue enrichment analysis of genes of interest.

### Immunofluorescence and microscopy

Flies were briefly washed with 95% ethanol then dissected in PBS to isolate intestines. Samples were fixed for 30 min at room temperature in 4% formaldehyde. Samples were quickly washed in PBS+0.3% Triton-X (PBT), followed by three 10 min washes in PBT. Samples were blocked for 1 h in PBT+3% bovine serum albumin (BSA) at room temperature, then incubated overnight at 4°C in PBT+3% BSA with 1° anti-Cora 1:100 (DSHB, C615.16). Samples were washed three times for 10 min in PBT, then incubated for 1 h at room temperature with 2° Alexa anti-mouse 1:500. Samples were briefly washed with PBT, followed by three 10 min washes in PBT. Hoechst DNA stain 1:500 was added to the second 10 min wash. Samples were washed in PBS, then mounted on slides in Fluoromount (Sigma-Aldrich F4680).

Slides were visualized on a spinning disk confocal microscope (Quorum WaveFX; Quorum Technologies Inc). The R4/R5 region of the posterior midgut of each sample was located by identifying the midgut–hindgut transition and moving one or two frames anterior from the attachment site of the Malpighian tubules. Images were acquired using Velocity Software (Quorum Technologies). Three-dimensional reconstruction was performed with Icy.

### Quantification of coracle

Quantification of localization of coracle in images was performed in FIJI. Three representative cells were selected per 40X image. For each cell, a transverse line was drawn across the bicellular junction into the cell to measure coracle expression. Peak expression was recorded as the junction value and 2.24 µm (10 px) into the cell from this peak level was recorded as the cytosol value. The junction/cytosol ratio was calculated from these two values. This was performed in triplicate for each cell, and the average of these three measurements was recorded as the value for the cell. Sample sizes for flies raised on HF (*n*=7 guts, 66 cells) and GSF (*n*=8 guts, 84 cells).

### Barrier function assays

For the smurf assay, HF and GSF were prepared as described with the addition of 1% erioglaucine disodium salt (Brilliant Blue FCF). Flies were raised on their respective diets and monitored daily for extraintestinal leakage of dye or ‘smurfing’. For the dextran sulphate sodium (DSS) challenge, flies were raised on either HF or GSF for 20 days, then transferred to either HF or GSF with 5% DSS added, respectively. Deaths were recorded daily and flies were transferred to fresh food every 2–3 days.

### Statistical analysis

Statistical analysis was performed using Graphpad Prism (Version 7.0). Statistical significance was set at *P*<0.05. Significance between two samples was determined by Student's *t*-tests. Significance in experiments with two independent variables were determined by two-way analysis of variance (ANOVA). For lifespan and survival analysis, significance was determined using log-rank (Mantel-Cox) test. Hazard function was determined with 5 day bins.

## Supplementary Material

Supplementary information
