# Supplementary material for: A glucose-supplemented diet enhances gut barrier integrity in Drosophila
Source: Biol Open. 2021 Mar 8;10(3):bio056515. doi: 10.1242/bio.056515 (PMC7969588; doi:10.1242/bio.056515)
Supplement: Supplementary information [file biolopen-10-056515-s1.pdf]

Table S1.

Components of holidic food. The holidic medium was prepared following the published protocol and recipe using the original AA solution (Oaa) at 100mM biologically available nitrogen (Piper *et al.* 2014). Amounts listed are per 1 litre of food. Supplementation to the unmodified recipe is described.

RECIPE	COMPONENTS	AMOUNT (g or mL)
Unmodified (HF)	agar	20
	sucrose	17.12
	cholesterol	0.30
	abs. ethanol	15
	acetic acid	3
	KH2PO4	3
	NaHCO3	1
	CaCl2	0.25
	MgSO4	0.25
	CuSO4	0.0025
	FeSO4	0.025
	MnCl2	0.001
	ZnSO4	0.025
	choline chloride	0.05
	myo-inositol	0.005
	inosine	0.065
	uridine	0.06
	thiamine	0.0014
	riboflavin	0.0007
	nicotinic acid	0.0084
	Ca pantothenate	0.0108
	pyridoxine	0.0017
	biotin	0.0001
	folic acid	0.0005
	L-isoleucine	0.91
	L-leucine	0.605
	L-tyrosine	0.21
	L-phenylalanine	0.393
	L-histidine	0.303
	L-lysine	0.575
	L-methionine	0.242
	L-arginine	0.242
	L-threonine	0.605
	L-valine	0.847
	L-tryptophan	0.151
	L-alanine	1.059
	L-cytosine	0.015
	L-aspartic acid	0.514
	L-glycine	0.968
	L-asparagine	0.514
	L-proline	0.454
	L-glutamine	0.757
	L-serine	0.575
	L-glutamic acid	0.757
	propionic acid	6
	nipagin	15
	water	Bring up to 1 L
**Recipes below include the above components plus the listed supplementation**		
Glucose-supplemented (GSF)	D-glucose	50
Casein-supplemented	casein	50
Lard-supplemented	lard	22.2

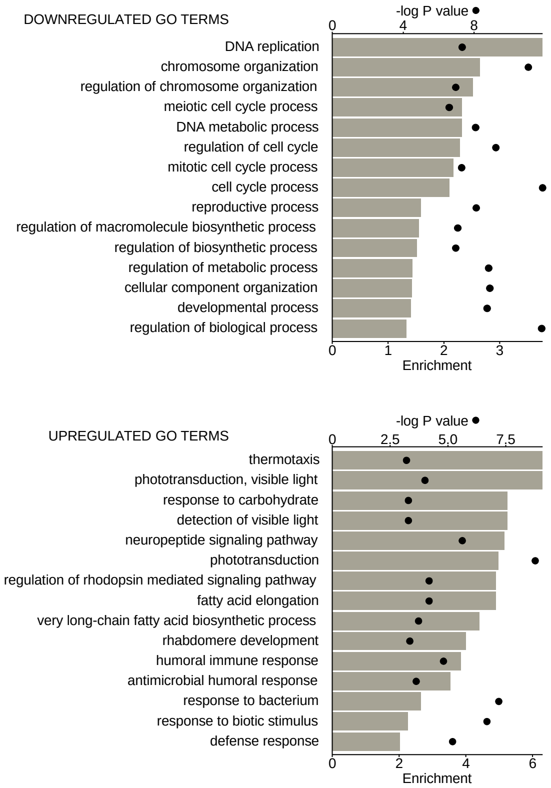

FIGURE S1

Figure S1. Microarray comparison of female flies raised on glucose-supplemented versus unmodified food.

Gene Ontology (GO) analysis from down- or up-regulated differentially expressed genes from comparison of female flies raised on glucose-enriched food (100 g/L) versus unmodified holidic food. Bars (bottom x axis) represent enrichment scores and black circles (top x axis) represent -logP values for each enriched GO term.

Biology Open • Supplementary information

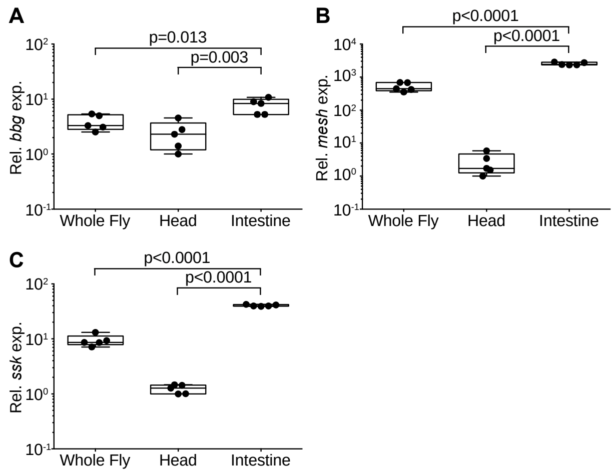

FIGURE S2

Figure S2. Tissue expression of cell junction genes.

(A-C) Quantification of the relative expression of (A) *bbg*, (B) *mesh*, and (C) *ssk* in whole flies, heads, or intestines from 20-day old w^{1118} flies raised on unmodified holidic food (HF). Statistical significance determined by Student's T-test.

Biology Open • Supplementary information
